# Key ethical considerations to guide the adjudication of a single-dose HPV vaccine schedule

**DOI:** 10.1080/21645515.2021.1917231

**Published:** 2021-05-19

**Authors:** Ruha Shadab, James V. Lavery, SarahAnn M. McFadden, Jad A. Elharake, Fauzia Malik, Saad B. Omer

**Affiliations:** a Yale Institute for Global Health, New Haven, CT, USA; b Hubert Department of Global Health, Rollins School of Public Health and Center for Ethics, Emory University, Atlanta, GA, USA; c Department of Internal Medicine, Infectious Disease, Yale School of Medicine, New Haven, CT, USA; d Department of Health Policy and Management, Yale School of Public Health, New Haven, CT, USA; e Yale School of Nursing, Orange, CT, USA; f Department of Epidemiology of Microbial Diseases, Yale School of Public Health, New Haven, CT, USA

**Keywords:** HPV vaccine ethics, policy, distributive justice, single-dose schedule

## Abstract

There is a high burden of human papillomavirus (HPV) associated cancers in low- and middle-income countries (LMICs). Reducing the recommended dosing schedule from two doses to one makes the vaccine schedule logistically simpler and lowers the cost. This could make the distribution of the current vaccine supply more equitable and lead to the protection of more people. However, the clinical trials studying the efficacy of a single-dose schedule have not yet delivered final results. Against this background, the question is whether a single-dose HPV vaccine recommendation is appropriate now, and if so, what are the ethical considerations of such a recommendation? We developed three ethical recommendations: (1) adopt a holistic view of evidence to justify policy decisions; (2) prioritize the reduction in global disparities in decision-making at all levels; and (3) be transparent in the reporting of how key stakeholder interests have shaped the collection and interpretation of the evidence, and ultimate decisions. The complex discussion regarding the HPV single-dose vaccine schedule highlights the need for in-depth engagement globally to improve our understanding of country-specific contexts, and how those contexts influence decisions regarding the HPV vaccine single-dose recommendation.

In 2014, the World Health Organization (WHO) issued a recommendation that National Immunization Programs (NIPs) should adopt a two-dose schedule of the human papillomavirus (HPV) vaccine for children 9 to 14 years of age,^1^ based on the non-inferior antibody response in this age group compared to 16 to 26 year old women in the phase 3 clinical trials.^1–3^ Four years later, in May 2018, WHO issued a call for action toward global cervical cancer elimination.^4^ This is estimated to increase the total demand for HPV vaccines to 120 M doses or more per year after 2025. Sizable increases in supply will be required to serve this level of demand. Supply constraints are expected until at least 2023–24.^5^ Moreover, some promising findings of the potential efficacy of a single dose of the HPV vaccine^6–8^ have initiated a debate within the global vaccine community about the desirability of moving from the two-dose schedule to a single-dose schedule.

The consideration of a single-dose schedule occurs against the backdrop of staggering global disparities in HPV vaccine coverage^9–11^ and cervical cancer incidence and prevalence between high-income countries (HICs) and low- and middle-income countries (LMICs).^12^ Although appeals to “equity” are common in global health discussions, precisely how deliberations about ethics should be structured to help guide decisions about the single-dose HPV vaccine have not received adequate attention. The annual global birth cohort of girls is currently 60 million, only 10 million of whom are currently being vaccinated, mainly due to the lack of HPV vaccine offerings in LMICs.^9^ Part of the WHO’s strategy to eliminate cervical cancer as a public health problem includes 90% of girls being fully vaccinated with the HPV vaccine by the age of 15 years.^11^ Furthermore, less than 5% of eligible girls in LMICs are vaccinated, even though LMICs account for ~90% of the cases of cervical cancer worldwide.^13^ In this paper, we present the findings of our investigation into the ethics of the HPV vaccine single-dose debate. First, we present our review and critical appraisal of the literature. Then, we present a set of recommendations distilled from these analyses. The recommendations are intended to help individuals and organizations intimately involved in the single-dose debate to frame the ethical implications of their deliberations and to encourage explicitness in the justification of decisions for or against the approval of the single-dose schedule.

We reviewed and critically analyzed the literature pertaining to the scientific rationale for a single-dose HPV vaccine. In the absence of a clear correlate of immunity to HPV infection, and with initial evidence of improved immune response after second priming and third boost doses, HPV vaccine development has followed a “classic” prime-boost dosing model, settling, initially, on a three-dose schedule. This approach was also deemed most likely to confer the longest duration of protective immunity, compared to shorter dose schedules.^14^ Therefore, in 2009, the WHO recommended that countries adopt a three-dose schedule for their National Immunization Programs.^15^

Trials of the three-dose HPV vaccine schedule have demonstrated consistently high levels of efficacy and effectiveness.^16^ However, due to incomplete dosing, or no doses in the case of those women assigned to placebo controls, post-hoc analyses from some of these trials have allowed comparisons of protective immunity in young women who received only two or one dose.^6,8,17^ Evidence from prospective non-inferiority immunogenicity trials^18^ prompted the WHO to recommend the two-dose schedule for girls who receive the first dose prior to 15 years of age^1^ and led both the Pan American Health Organization (PAHO) and the European Medicines Agency (EMA) to approve a two-dose schedule in 2014^19^ and 2015, respectively.^20^ Because the analyses of the performance of a single dose of vaccine, compared to 3, 2 and 0 doses, have been surprisingly strong,^7^ it has now turned the attention of the global vaccine community to the desirability and advisability of a single-dose HPV vaccine schedule, which could greatly improve the low HPV vaccine uptake in LMICs. These decisions will likely be made by the National Immunization Technical Advisory Groups (NITAGs) with input from Regional Immunization Technical Advisory Groups (RITAGS); therefore, scientific evidence and ethical considerations need to be easily assessible and clear to help countries/regions improve HPV vaccine coverage and reduce the burden of HPV-associated cancers.

The rationales for exploring the relative efficacy of a single-dose schedule compared to the current WHO-recommended two-dose schedule^1^ have focused on four main considerations: (1) evidence regarding relative immunogenicity and durability of protective immunity; (2) elucidation of immunological mechanisms of protective immunity; (3) potential for increased coverage; and (4) potential for lower schedule and program cost and improved cost-effectiveness.

First, we discuss the relative immunogenicity and durability of protective immunity. Despite a rich and extensive literature about all aspects of HPV vaccines, very few studies have been designed explicitly to compare three-dose vaccines with two or fewer doses. Numerous ongoing studies have reported encouraging efficacy data for a single-dose, but these have all been post-hoc analyses.^6,7^ A randomized control trial (RCT) addressing the comparative efficacy of single-dose vs. two-dose HPV vaccine schedules is due to report findings in 2024–25.^7,21^

Published comparisons of two-dose schedules with one-dose schedules are drawn primarily from retrospective analyses of post-licensure studies from national immunization programs, nested observational studies of random allocations in clinical trials, immunogenicity studies, and case-control and other observational studies from national registries and commercial health systems data.^22–25^

Evidence from these sources is strongly suggestive that a single-dose schedule is capable of conferring protective immunity and that immunity lasts up to 11 years,^26^ but limitations in the available data have made it extremely difficult to assess non-inferiority compared to three- and two-dose schedules. Additionally, there are several different HPV vaccines, and durable protection from a specific bivalent vaccine may not necessarily transfer to other formations, such as the quad- or non-valent. The implications of these immunological differences for long-term clinical protection remain unclear. Additionally, because the prospectively designed and controlled comparative trials have yet to report their results, the true implications of these findings for vaccine effectiveness against HPV infection and related cancers are difficult to assess. These uncertainties led the SAGE to recommend to the WHO in 2018 that there is currently insufficient evidence to support a determination of non-inferiority of a single-dose schedule.^11^

Second, we critically evaluate the immunological mechanisms that support a single-dose HPV vaccine schedule. Schiller and Lowy recently summarized the current immunology of HPV vaccination and concluded that “(t)here is mounting evidence that the vaccines have similar efficacy and effectiveness even when administered in a single dose.”^27^ Their review highlights the unique morphology of the vaccine’s virus-like particles (VLPs), which self-assemble from 360 individual copies of the L1 major capsid protein of the virus. The structure of the VLPs is just one of the complexities of the vaccine manufacturing process. The L1 VLPs display a densely and highly ordered arrangement of epitopes that present a “pathogen-specific danger signal” [p. 4770],^27^ common in a wide range of viruses and microbial surfaces. This unique structure makes the VLPs highly effective in causing the proliferation of B cells and antibody-producing long-lasting plasma cells (LLPCs) in particular, which appear to ensure a stable and durable source of ongoing antibody production. Schiller and Lowy address the concern that a single-dose vaccine appears to produce lower levels of antibody than multiple doses by speculating that “the observation that antibody levels that are more than 100-fold lower than the minimum level detected in the *in vitro* neutralizing assay are able to prevent *in vivo* infection is consistent with the idea that there are potent antibody-mediated mechanisms relevant to *in vivo* inhibition that are not detected *in vitro*” [p. 4771].^27^ Although not determinative, the unique immunological dynamics of the HPV vaccine offer strong biological plausibility for durable protective immunity from a single-dose HPV vaccine, an effect that may not be well reflected in the empirical evidence to date, which emphasizes the importance of antibody titers.

Third, we discuss the potential for increased HPV vaccine coverage. Approximately 90% of the 530,000 annual new cases of cervical cancer worldwide occur in LMICs, only about 5% of eligible women in LMICs have received HPV vaccination.^14^ Since the HPV vaccine is difficult to manufacture^28^ and relies on cold-chain delivery because of its relative heat-sensitivity,^28,29^ it is costly to produce and distribute. Improved coverage from a single-dose versus a two-dose schedule is the most intuitive of the prospective benefits of a switch to a single-dose schedule. However, more experience and data about distribution cost-savings will be required to quantify the potential increases in coverage that a single-dose policy is likely to produce.^30^

Finally, we explored the literature about a single-dose HPV vaccine and the potential for lower costs and improved cost-effectiveness. Compared to a two-dose HPV vaccine schedule, a single-dose should reduce logistical and administration costs per person vaccinated for NIPs. However, overall costs to NIPs in LMICs could increase if a single-dose schedule facilitates a significant expansion of overall coverage beyond the current 5%. The cost-effectiveness of the single-dose policy will ultimately be determined by the vaccine’s effectiveness at creating and sustaining individual and herd immunity, and by its impact on reducing the incidence of target cancers.^31^

In 2019, SAGE recommended that “countries that have already introduced HPV vaccine and face an imminent vaccine supply shortage can consider a 1 + 1 schedule.” A “1 + 1 schedule” is also called an “extended interval” schedule, where the first dose is given to girls aged 9–10 years, and the second dose is given 3 to 5 years later. The implementation of a 1 + 1 schedule will require robust monitoring and surveillance in each implementing country to guide decisions about the need for a second dose. In theory, cost-savings from delayed purchase of the second dose at the outset could be applied to creating and/or strengthening the necessary surveillance capacity, which could support other vaccine and disease programs as well.

After reviewing the literature, we now discuss key ethical issues and recommend an ethical framework for the adjudication of a single-dose HPV vaccine schedule. The choice of HPV vaccine schedule raises ethical challenges for reasons of disparity in access, supply constraints, high cost, delivery complexity, and limitations of the existing scientific evidence. Decision makers, especially those at the country or regional levels, are faced with three key ethical questions:

1. **Zero versus one dose**: Should a country that has not yet rolled out an HPV vaccination program, begin with a one-dose schedule?
2. **Two doses versus one dose**: Should countries that already have a two-dose HPV vaccination schedule in place move to a one-dose schedule?
3. **Fair interpretation of limited evidence**: What inferences can policymakers make from the existing scientific evidence? And more specifically, how well does technical inferiority in immunogenicity and/or efficacy predict the relevant public health impact of a single-dose schedule?

With this understanding of the ethical issues and biological plausibility of a single-dose HPV vaccine schedule, we now introduce an ethical framework to help guide policy-makers, at all levels, facing these ethical challenges. The overarching ethical question facing the global vaccine community is how best to protect an entire generation of young women in LMICs from devastating and highly preventable cancers. Reduction in the per person cost of vaccination could facilitate expanded population coverage in those countries with the lowest coverage rates, which could provide a realistic pathway to reducing global disparities in HPV infection and cancer prevalence. Intra-country disparities are also common in HPV vaccine distribution. Although it is unclear to what extent these disparities are driven primarily by cost as opposed to supply chain deficiencies and unfair distribution policies, cost reduction could offer new possibilities for improving the fairness of distribution within countries, in addition to expanded coverage. These are, fundamentally, and unavoidably, questions of global justice, both in absolute terms – that is, whether entire generations of young women (and young men) are simply denied access to a life-saving intervention – and in relative terms – that is, whether birth cohorts of young women (and young men) in HICs continue to reap a disproportionate share of the benefits of HPV vaccination compared to their counterparts in LMICs.

The complex scientific questions outlined above also present difficult ethical questions about the appropriate standards of evidence for justifying decisions to recommend a single-dose policy, or not, or to endorse a 1 + 1 approach, or to support multiple-dose schedules simultaneously. These policy decisions involve the navigation of complex and imperfect scientific evidence alongside key contextual considerations, such as affordability and different coverage and programmatic goals among NIPs.^32^

Conventional appeals to ethical principles and guiding values are likely to be inadequate to provide meaningful ethical guidance for all the relevant stakeholders, including manufacturers, researchers, regulators, normative bodies, national immunization programs, host country policymakers and health systems administrators, and vaccine funding and purchasing bodies. In the face of these challenges, we propose a set of key ethical considerations that can function as an “ethics roadmap” to help shape an approach to ethical reasoning that is ‘fit for purpose’ for the complex ethical challenges that must be navigated during the period of evaluation and possible transition to a single-dose regimen. We focus, in particular, on three key recommendations: (1) adopt a holistic view of evidence to justify policy decisions; (2) prioritize the reduction in global disparities in decision-making at all levels; and (3) be transparent in the reporting of how key stakeholder interests have shaped the collection and interpretation of the evidence, and ultimate decisions.

The first recommendation is to adopt a holistic view of the evidence to justify policy decisions. The evidence, described above, informing the debate about the relative effectiveness of a single-dose HPV vaccine schedule is impressive and compelling and will become more complete over time. One ethical hazard associated with this evidence landscape is that decision-makers may, by convention, adopt an overly narrow analytic frame that relies predominantly on measures like the persistence of neutralizing antibodies over time, even though our understanding of the precise relationship between antibody titers and protective immunity for HPV vaccines is incomplete.^27^ There is no debate about the relevance or importance of antibody titers as a signal of immune response and likely protective immunity. However, it is conceivable that decision-makers could find the single-dose schedule to be inferior to the two-dose schedule on the basis of this incomplete knowledge, and, in doing so, inadvertently establish a “double standard” whereby LMICs may avoid the adoption of the vaccine, or the expansion of their coverage, to avoid having to justify the adoption of a ‘lower standard.’^33^

Although there are no simple solutions to evidence that is restricted to efficacy, we recommend that decision-makers adopt a holistic view of what evidence can be useful to support robust policy decisions. More explicitly factoring in considerations of cost and delivery logistics, for example, as relevant features of the ‘performance’ of the single-dose schedule could help to expand the working concept of “effectiveness” beyond the achievement of primary and secondary endpoints, to broader population goals of improved coverage and perhaps even improved immunization infrastructure.^32^ To do so might a more explicit “on balance” assessment of how the relevant features of the single-dose schedule might combine to produce a unique value profile, beyond efficacy alone.^34^

The second recommendation is to prioritize the reduction of global disparities. One implication of adopting an excessively narrow interpretation of effectiveness, as described above, is that any decision that establishes a single-dose schedule as inferior to the two-dose schedule, in absolute terms, is unlikely to be overturned until the current Costa Rica RCT comparing single to two-dose regimens reports in 2024–2025.^7,21^ In the meantime, uptake of a two-dose schedule (i.e., the ‘superior’ schedule) would likely remain inaccessible to low-income, and lower-middle-income countries due to cost considerations. Therefore, whatever reductions in uncertainty the Costa Rica trial might provide, will almost certainly be felt in a widening of global disparities. Thus, the opportunity for expanding immunization capacity and/or coverage that the single-dose schedule could provide for some countries would be delayed for another 5 years. This scenario would privilege individual-level gains *within* countries rather than relative gains *between* and *among* countries.

John Rawls’s theory of justice addresses the issue of fair distribution of social goods.^35^ He contends that society is conceived of as a fair system of cooperation from one generation to the next between free and equal citizens possessed of the two moral powers, which are: (1) the capacity to form, revise, and rationally act upon a conception of the good; and (2) the capacity for a sense of justice. In the current controversy over whether the potential transition to a single-dose HPV vaccine schedule is warranted scientifically, Rawls’s framing makes explicit the importance of conceptualizing and generating evidence in a way that clarifies the nature and specific value of the social goods in question. Although relatively few LMICs have introduced the HPV vaccine into their national immunization programs, the coverage achieved in these countries has been high.^36^ It would be unfair if the potential reductions in global disparities that this increased coverage represents were excluded from consideration simply by the conventions of RCT data collection and analysis.

The final recommendation is the need for transparency regarding the influence of stakeholder interests. Many national introductions of the HPV vaccine in LMICs were funded by pharmaceutical companies’ donations.^9^ This changed in 2013 when LMICs were able to receive support for HPV vaccine programs through Gavi, the Vaccine Alliance (formally known as the Global Alliance for Vaccines and Immunizations).^37^ The critical determinants of effectiveness, evidentiary standards and the potential social value of vaccines, or candidate vaccines are routinely discussed by stakeholders within the complex global vaccine enterprise, including companies, public and private funders, regulators, Gavi, WHO, individual target country ministries, and national immunization programs. But although the discussions are well known to involve complex consultations and negotiations, the ways in which the interests of these stakeholders shape decisions – beyond the technical interpretation of the available data – are rarely made explicit or publicly accessible. This is important because these stakeholders’ interests can carry significant weight, particularly in individual country decisions, and could distort the ethical intentions reflected in other aspects of the decision-making process, including the potential to undermine the priority of reducing global disparities.

All decisions have implications. The primary function of scientific evidence is to reduce our uncertainty about the nature of the phenomena we study and, by doing so, increase the confidence we can have in the decisions that evidence aims to inform. Although there is a common understanding that values play a critical role in decision-making, there is limited acknowledgment that values can play a critical role in the interpretation of evidence.^38^ Upshur has argued that “inherent to the interpretation of evidence are very different animating values.”^38^ He describes two forms of prudence – “active” and “precautionary” – that lean toward pragmatism and beneficence, and non-maleficence, respectively.

Given the current state of the evidence, described above, if a country decides to launch a new HPV vaccination program with a single-dose schedule, or switch from a two-dose to a single-dose schedule, it seems likely that the “animating value” motivating that decision would be a desire to maximize its ability to protect its young women (and young men) from preventable cancers. Such a decision would not constitute a disregard of the current evidence, but rather an interpretation favoring various elements of the evidence, e.g., the strong biological plausibility that a single dose can durably stimulate antibody production, and thereby sustain protective immunity, and that the single-dose schedule represents a significant advantage in terms of feasibility and affordability. This type of “active prudence” reflects the ethical burden that country-level decision-makers shoulder in terms of stewardship of resources and the accountabilities of a government to its people, in contexts that are experiencing dramatic increases in preventable cancers.

On the other hand, policy-developers, such as SAGE members as well as national and regional immunization technical advisory groups, are charged not only with interpreting the available evidence about safety and efficacy and likely effectiveness but also with the broader mandate of protecting the scientific and evidentiary standards themselves. From this perspective, Upshur’s notion of “precautionary prudence” might be best understood as a defense against interpretations of available evidence that might lead to the dilution of, or manipulation of, evidentiary standards.

The challenge facing all decision-makers with respect to the adjudication of a single-dose HPV vaccine regimen is to ensure that these different forms of ‘prudence’ on the part of decision-makers do not result in millions of young women (and young men) being denied the opportunity for protection against preventable cancers because we lack sufficiently sensitive procedures for integrating value judgments with the best available evidence. Although our ethical framework is designed to be flexible with broad inter-country application, it cannot account for all factors that decision-makers must consider. However, the framework was intentionally designed not to be prescriptive or exhaustive of all the potentially relevant factors, but instead to provide decision-makers with a set of considerations that should be taken into account to ensure that ethical considerations – including significant implications for global justice – are not inadvertently discounted in their decision-making.

This paper presents an overview of ethical considerations for policymakers engaged in deliberations and decision-making about the status of a single-dose HPV vaccine schedule. Specifically, we offer a “roadmap” of three ethical considerations (Table 1) that we believe offer a constructive set of navigation aids to help keep ethical considerations tightly linked to the adjudication of emerging scientific evidence and contextual considerations.^32^ These ethical parameters encourage a healthy tension between narrow interpretations of efficacy and effectiveness and broader considerations of the potential social value of a single-dose schedule, against the backdrop of crushing global disparities in HPV and cancer prevalence between HICs and LMICs.

**Table 1.** Key ethical parameters

(1)Adopt a holistic view of evidence to justify policy decisions
(2)Prioritize the reduction of global disparities
(3)Transparency regarding the influence of stakeholder interests

## References

[1] World Health Organization. Comprehensive cervical cancer control: A guide to essential practice. 2nd ed. Geneva: World Health Organization Press; 2014. 25642554

[2] Iversen O-E, Miranda MJ, Ulied A, Soerdal T, Lazarus E, Chokephaibulkit K, Block SL, Skrivanek A, Nur Azurah AG, Fong SM, et al. Immunogenicity of the 9-valent HPV vaccine using 2-dose regimens in girls and boys vs a 3-dose regimen in women. Jama. 2016;316(22):2411–6. doi:10.1001/jama.2016.17615. 27893068

[3] Romanowski B, Schwarz TF, Ferguson L, Peters K, Dionne M, Behre U, Schulze K, Hillemanns P, Suryakiran P, Thomas F, et al. Sustained immunogenicity of the HPV-16/18 AS04-adjuvanted vaccine administered as a two-dose schedule in adolescent girls: five-year clinical data and modeling predictions from a randomized study. Hum Vaccin Immunother. 2016;12(1):20–29. doi:10.1080/21645515.2015.1065363. 26176261 PMC4962738

[4] World Health Organization. Executive board, session 144; document EB144/28. 2019 Jan 30.

[5] World Health Organization. Global market study: HPV. 2019.

[6] Brotherton JM, Budd A, Rompotis C, Bartlett N, Malloy MJ, Andersen RL, Coulter KA, Couvee PW, Steel N, Ward GH, et al. Is one dose of human papillomavirus vaccine as effective as three?: a national cohort analysis. Papillomavirus Res (Amsterdam, Netherlands). 2019;8:100177. doi:10.1016/j.pvr.2019.100177. 31319173 PMC6658930

[7] Kreimer AR, Herrero R, Sampson JN, Porras C, Lowy DR, Schiller JT, Schiffman M, Rodriguez AC, Chanock S, Jimenez S, et al. Evidence for single-dose protection by the bivalent HPV vaccine—Review of the Costa Rica HPV vaccine trial and future research studies. Vaccine. 2018;36(32):4774–82. doi:10.1016/j.vaccine.2017.12.078. 29366703 PMC6054558

[8] Kreimer AR, Rodriguez AC, Hildesheim A, Herrero R, Porras C, Schiffman M, González P, Solomon D, Jiménez S, Schiller JT, et al. Proof-of-principle evaluation of the efficacy of fewer than three doses of a bivalent HPV16/18 vaccine. J Natl Cancer Inst. 2020;8(2):e191–e203. doi:10.1093/jnci/djr319. PMC3186781 21908768

[9] Gallagher KE, LaMontagne DS, Watson-Jones D. Status of HPV vaccine introduction and barriers to country uptake. Vaccine. 2018;36(32):4761–67. doi:10.1016/j.vaccine.2018.02.003. 29580641

[10] LaMontagne DS, Bloem PJN, Brotherton JML, Gallagher KE, Badiane O, Ndiaye C. Progress in HPV vaccination in low- and lower-middle-income countries. Int J Gynaecol Obstet. 2017;138(Suppl 1):7–14. doi:10.1002/ijgo.12186. 28691329

[11] World Health Organization. Working group on potential contribution of Human Papillomavirus (HPV) vaccines and immunization towards cervical cancer elimination: background document and report to SAGE. 2019.

[12] Arbyn M, Weiderpass E, Bruni L, de Sanjosé S, Saraiya M, Ferlay J, Bray F.Estimates of incidence and mortality of cervical cancer in 2018: a worldwide analysis. Lancet Glob Health. 2020;8(2):e191–e203. doi:10.1016/S2214-109X(19)30482-6. 31812369 PMC7025157

[13] Ginsburg OM. Breast and cervical cancer control in low and middle-income countries: human rights meet sound health policy. J Cancer Policy. 2013;1(3–4):e34–e41. doi:10.1016/j.jcpo.2013.07.002.

[14] Stanley M, Dull P. HPV single-dose vaccination: impact potential, evidence base and further evaluation. Vaccine. 2018;36(32Pt A):4759–60. doi:10.1016/j.vaccine.2018.02.076. 29754700

[15] World Health Organization. WHO position on HPV vaccines. Vaccine. 2009;27(52):7236–37. doi:10.1016/j.vaccine.2009.05.019. 19450645

[16] Schiller JT, Castellsagué X, Garland SM. A review of clinical trials of human papillomavirus prophylactic vaccines. Vaccine. 2012;30(Suppl 5(5)):F123–138. doi:10.1016/j.vaccine.2012.04.108. 23199956 PMC4636904

[17] Sankaranarayanan R, Joshi S, Muwonge R, Esmy PO, Basu P, Prabhu P, Bhatla N, Nene BM, Shaw J, Poli URR, et al. Can a single dose of human papillomavirus (HPV) vaccine prevent cervical cancer? Early findings from an Indian study. Vaccine. 2018;36(32):4783–91. doi:10.1016/j.vaccine.2018.02.087. 29551226

[18] Donken R, Knol MJ, Bogaards JA, van der Klis FR, Meijer CJ, De Melker HE. Inconclusive evidence for non-inferior immunogenicity of two- compared with three-dose HPV immunization schedules in preadolescent girls: a systematic review and meta-analysis. J Infect. 2015;71(1):61–73. doi:10.1016/j.jinf.2015.02.005. 25709084

[19] Pan American Health Organization Technical Advisory Group. Technical Advisory Group on vaccine-preventable diseases (TAG). 2014.

[20] European Medicines Agency. Annex 1: summary of product characteristics. 2015.

[21] Scientific evaluation of one or two doses of the bivalent or nonavalent prophylactic HPV vaccines. [accessed 2020 August 10]. https://ClinicalTrials.gov/show/NCT03180034

[22] Markowitz LE, Drolet M, Perez N, Jit M, Brisson M. Human papillomavirus vaccine effectiveness by number of doses: systematic review of data from national immunization programs. Vaccine. 2018;36(32):4806–15. doi:10.1016/j.vaccine.2018.01.057. 29802000

[23] Whitworth HS, Gallagher KE, Howard N, Mounier-Jack S, Mbwanji G, Kreimer AR, Basu P, Kelly H, Drolet M, Brisson M, et al. Efficacy and immunogenicity of a single dose of human papillomavirus vaccine compared to no vaccination or standard three and two-dose vaccination regimens: a systematic review of evidence from clinical trials. Vaccine. 2020;38(6):1302–14. doi:10.1016/j.vaccine.2019.12.017. 31870572

[24] Markowitz LE, Hariri S, Lin C, Dunne EF, Steinau M, McQuillan G, Unger ER. Reduction in human papillomavirus (HPV) prevalence among young women following HPV vaccine introduction in the United States, National health and nutrition examination surveys, 2003–2010. J Infect Dis. 2013;208(3):385–93. doi:10.1093/infdis/jit192. 23785124

[25] Sankaranarayanan R, Prabhu PR, Pawlita M, Gheit T, Bhatla N, Muwonge R, Nene BM, Esmy PO, Joshi S, Poli URR, et al. Immunogenicity and HPV infection after one, two, and three doses of quadrivalent HPV vaccine in girls in India: a multicentre prospective cohort study. Lancet Oncol. 2016;17(1):67–77. doi:10.1016/S1470-2045(15)00414-3. 26652797 PMC5357737

[26] Tsang SH, Sampson JN, Schussler J, Porras C, Wagner S, Boland J, Cortes B, Lowy DR, Schiller JT, Schiffman M, et al. Durability of cross-protection by different schedules of the bivalent HPV vaccine: the CVT trial. J Natl Cancer Inst. 2020;112(10):1030–37. doi:10.1093/jnci/djaa010. 32091596 PMC7566371

[27] Schiller J, Lowy D. Explanations for the high potency of HPV prophylactic vaccines. Vaccine. 2018;36(32):4768–73. doi:10.1016/j.vaccine.2017.12.079. 29325819 PMC6035892

[28] Kumar S, Biswas M, Jose T. HPV vaccine: current status and future directions. Med J Armed Forces India. 2015;71(2):171–77. doi:10.1016/j.mjafi.2015.02.006. 25859081 PMC4388981

[29] Shank-Retzlaff ML, Zhao Q, Anderson C, Hamm M, High K, Nguyen M, Wang F, Wang N, Wang B, Wang Y, et al. Evaluation of the Thermal Stability of Gardasil®. Hum Vaccin. 2006;2(4):147–54. doi:10.4161/hv.2.4.2989. 17012891

[30] Burger EA, Campos NG, Sy S, Regan C, Kim JJ. Health and economic benefits of single-dose HPV vaccination in a Gavi-eligible country. Vaccine. 2018;36(32):4823–29. doi:10.1016/j.vaccine.2018.04.061. 29807710 PMC6066173

[31] Gallagher KE, Kelly H, Cocks N, Dixon S, Mounier-Jack S, Howard N, Watson-Jones D. Vaccine programme stakeholder perspectives on a hypothetical single-dose human papillomavirus (HPV) vaccine schedule in low and middle-income countries. Papillomavirus Res (Amsterdam, Netherlands). 2018;6:33–40. doi:10.1016/j.pvr.2018.10.004. 30352297 PMC6218645

[32] Upshur RE. Seven characteristics of medical evidence. Journal of Evaluation in Clinical Practice. 2000;6(2):93–97. doi:10.1046/j.1365-2753.2000.00244.x. 10970003

[33] Weijer C. The future of research into rotavirus vaccine. BMJ (Clinical Research Ed). 2000;321(7260):525–26. doi:10.1136/bmj.321.7260.525. 10968800 PMC1118429

[34] Freedman B. Equipoise and the ethics of clinical research. N Engl J Med. 1987;317(3):141–45. doi:10.1056/NEJM198707163170304. 3600702

[35] Rawls J. A theory of justice. Cambridge (MA): The Belknap Press of Havard University Press; 1971.

[36] Gallagher KE, Howard N, Kabakama S, Mounier-Jack S, Griffiths UK, Feletto M, Burchett HED, LaMontagne DS, Watson-Jones D. Lessons learnt from human papillomavirus (HPV) vaccination in 45 low- and middle-income countries. PloS One. 2017;12(6):e0177773. doi:10.1371/journal.pone.0177773. 28575074 PMC5456063

[37] The Vaccine GAVI Alliance. Millions of girls in developing countries to be protected against cervical cancer thanks to new HPV vaccine deals. 2013 [accessed 2020 Jun 9]. https://www.gavi.org/hpv-price-announcement.

[38] Upshur REG We need both evidence and values to navigate uncertainty. *Hastings Center Report*. 2014 Sept–Oct 4. 10.1002/hast.347 25231652

